# Supplementary material for: Identification of Critical Amino Acids of Coxsackievirus A10 Associated with Cell Tropism and Viral RNA Release during Uncoating
Source: Viruses. 2023 Oct 18;15(10):2114. doi: 10.3390/v15102114 (PMC10611408; doi:10.3390/v15102114)
Supplement: Supplementary file 1 [file viruses-15-02114-s001.zip › viruses-2646831-supplementary.pdf]

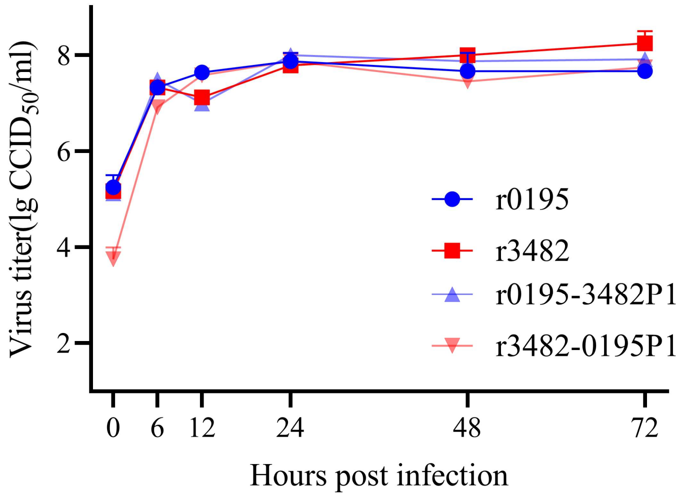

Figure S1. One-step growth curves for r0195, r3482, r0195-3482P1 and r3482-0195P1 in RD cells.

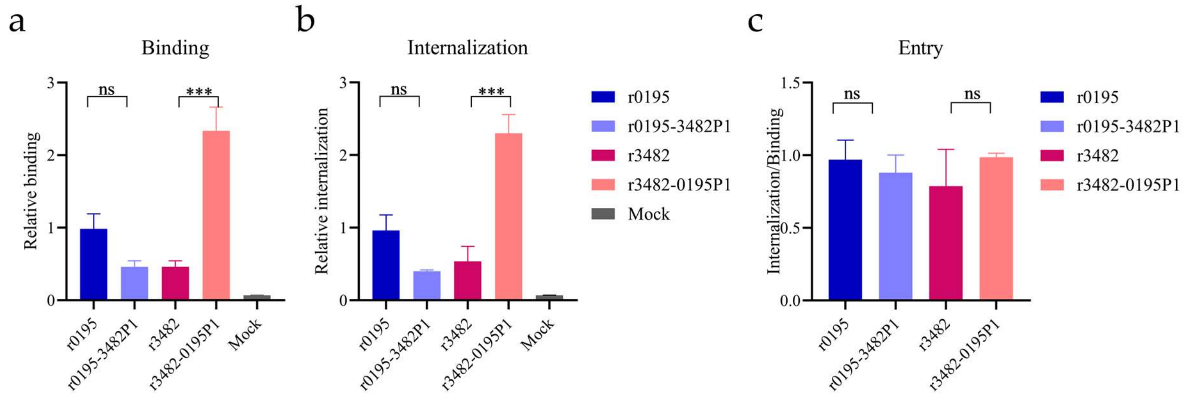

Figure S2. Binding and entry ability of r0195, r3482, r0195-3482P1 and r3482-0195P1 in RD cells. ***, $p < 0.001$, ns, no significance

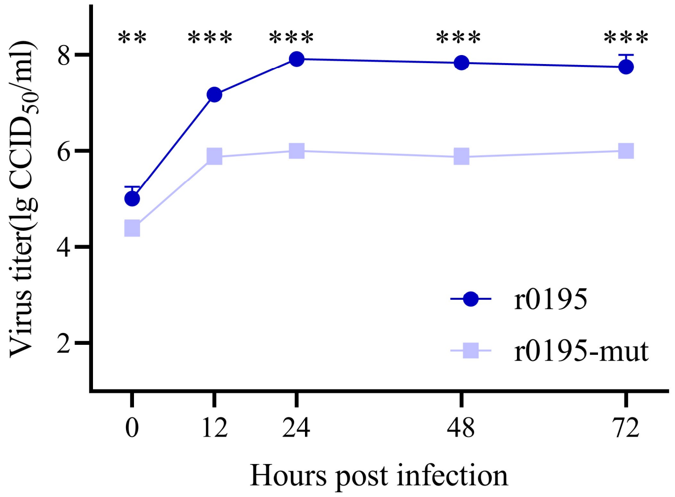

Figure S3. One-step growth curves for r0195 and r0195-mut in RD cells. **, $p < 0.01$; ***, $p < 0.001$.

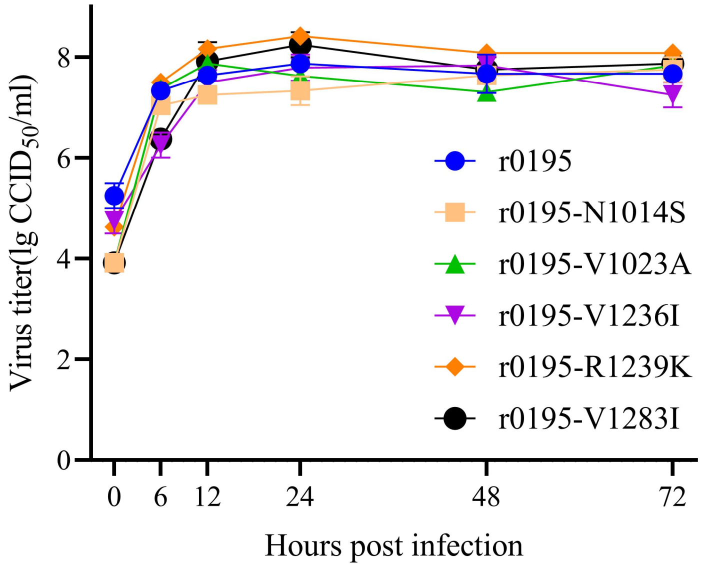

Figure S4. One-step growth curves for r0195 and r0195-mut in RD cells.
